# Case Report: Virus-induced rhabdomyolysis ranging from mild course to acute renal injury, hyperferritinemia and poor outcome: a case series of four pediatric patients

**DOI:** 10.3389/fped.2025.1552232

**Published:** 2025-05-09

**Authors:** Carlijn W. van der Zee, Giske Biesbroek, Michiel J. S. Oosterveld, Bart G. P. Koot, Nikki J. Schoenmaker, Taco W. Kuijpers

**Affiliations:** ^1^ Department of Pediatric Immunology, Rheumatology and Infectious Diseases, Emma Children's Hospital, Amsterdam University Medical Center, University of Amsterdam, Amsterdam, Netherlands; ^2^ Department of Pediatric Nephrology, Emma Children's Hospital, Amsterdam University Medical Center, University of Amsterdam, Amsterdam, Netherlands; ^3^ Department of Pediatric Gastroenterology, Emma Children's Hospital, Amsterdam University Medical Center, University of Amsterdam, Amsterdam, Netherlands; ^4^ Department of Pediatric Intensive Care, Emma Children's Hospital, Amsterdam University Medical Center, University of Amsterdam, Amsterdam, Netherlands

**Keywords:** virus-induced rhabdomyolysis, influenza A, hyperferritinemia, acute kidney injure (AKI), children

## Abstract

We report a case-series of four healthy pediatric patients presenting with virus-induced rhabdomyolysis. Rhabdomyolysis is characterized by rapid breakdown of skeletal muscle, which can result in mild complications to acute kidney injury and even death. In children rhabdomyolysis is often virus-induced. In this case series we describe two pediatric patients with severe complications, including acute kidney injury, hyperferritinemia and even in one case liver failure and death. The rhabdomyolysis in the other two patients was caused by different viruses and had a milder presentation and good outcome after hyperhydration. Our cases highlight the variability in clinical outcome in virus-induced rhabdomyolysis. The outcome of our cases may suggest that hyperferritinemia can play a critical role in the pathophysiology of acute kidney injury and therefore may be used as an important biomarker for disease severity.

## Introduction

Rhabdomyolysis is a clinical syndrome characterized by the rapid breakdown of skeletal muscle and release of its intracellular content into the systemic circulation. Rhabdomyolysis may result in acute kidney injury (AKI) partly due to direct toxic effect of myoglobin in the kidneys (1). Common causes of rhabdomyolysis in children are viral myositis, trauma, metabolic disorders, certain drugs and exercise (2). Especially in young children, more than one third of the rhabdomyolysis episodes is caused by viral infections (2). The severity of rhabdomyolysis varies from mild symptoms (myalgia, weakness and dark urine) to acute kidney injury and rarely death (1, 2).

Elevated ferritin levels are often reported in inflammatory conditions (3). Hyperferritinemia is characterized by ferritin levels >500 ng/dl (3, 4). Hyperferritinemia is often used as a biomarker to identify macrophage activation and is frequently elevated in hyperinflammatory syndromes, including cytokine storm syndromes, hemophagocytic lymphocytosis (HLH) and multi-inflammatory syndrome related to COVID-19. In children up to 40% of the inflammatory hyperferritinemia is caused by infections (3).

In this case series, we describe four pediatric patients who developed rhabdomyolysis secondary to a viral infection; two patients suffered from a complicated course of influenza A virus infection, one of a parainfluenza virus and enterovirus coinfection, and one of a primary cytomegalovirus (CMV) infection. In two cases, plasma ferritin levels were markedly increased. The aim of this report is to describe the severe complications of virus-induced rhabdomyolysis and strongly elevated ferritin levels in the more severe cases.

## Case descriptions

### Case A

**Figure 1:**
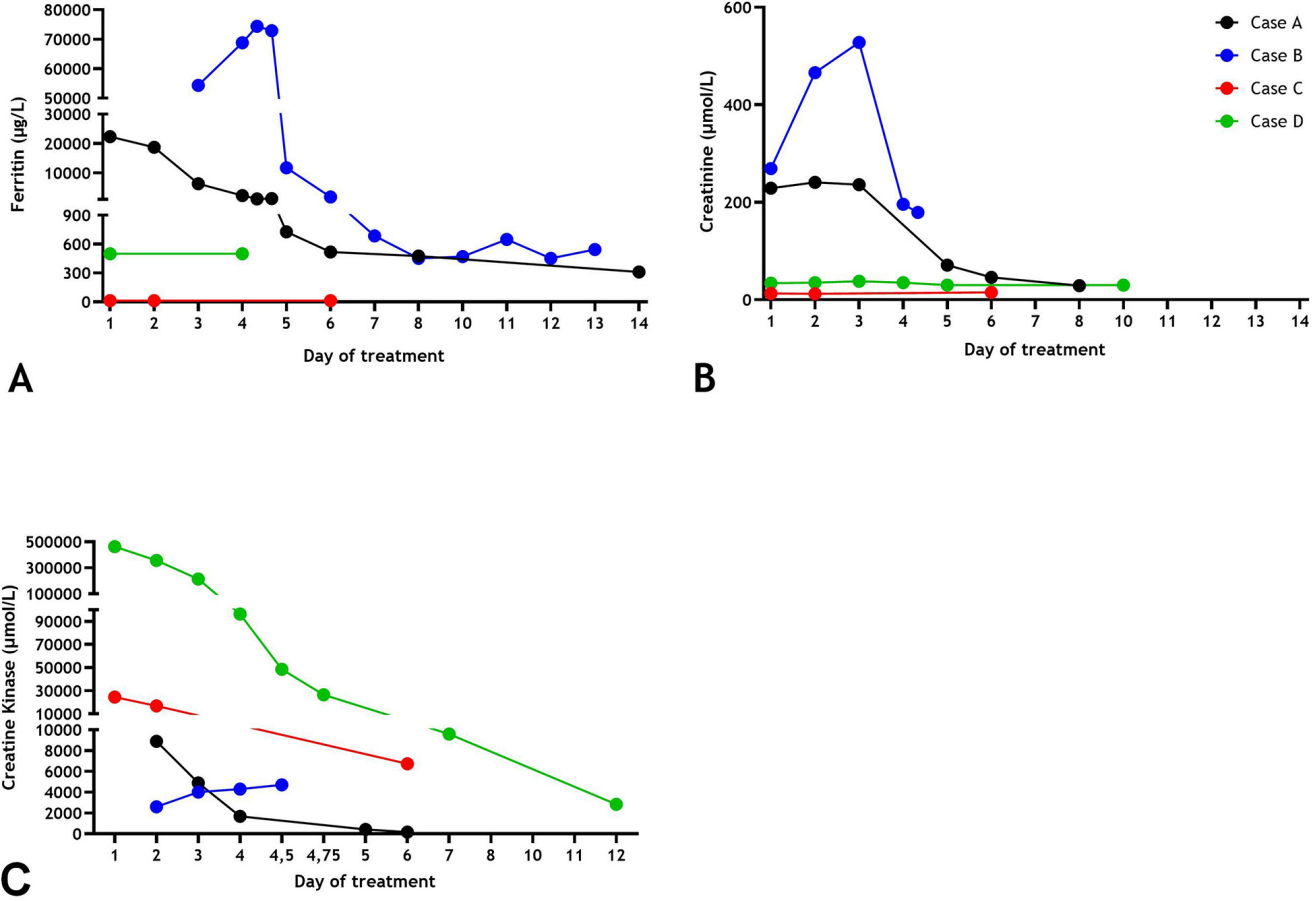
Serum ferritin **(A)**, creatinine levels **(B)**, creatinine kinase levels **(C)** over time of Cases **(A–D)**.

A 7-year-old boy was admitted to a local hospital after presenting with a history of pyrexia for 5 days, fatigue and pain around the left ear. Physical examination revealed cervical lymphadenopathy, skin rash and hepatomegaly. When he failed to respond to oral amoxicillin, the treatment was switched to intravenous ceftriaxone. On the subsequent day, the boy was transferred in stable condition to our center for further management of acute renal failure. Laboratory results are presented in Table 1. Striking findings were elevated levels of creatinine-kinase (Figure 1C), lactate dehydrogenase and transaminases (Table 1). Ferritin levels were markedly increased (Figure 1A). Urine analysis revealed hematuria and proteinuria. Since influenza A RNA was detected in a multiplex viral PCR test on the nasopharyngeal swab, antibiotic treatment was discontinued. The diagnosis was rhabdomyolysis and acute renal failure secondary to influenza A virus infection. Other causes of hyperferritinemic syndromes including neuroblastoma, hemophagocytosis and systemic juvenile idiopathic arthritis were excluded. Renal function improved with hyperhydration. After two weeks, the boy was discharged in good clinical condition. There was no clinical suspicion of an underlying immunodeficiency, as further substantiated by a whole exome sequencing (WES) and targeted analysis of a panel of 485 genes for inborn errors of immunity.

**Table 1:** Important laboratory findings during admission.

Virus	Case A	Case B	Case C	Case D	Normal values
	Influenza A	Influenza A	Parainfluenza Enterovirus	CMV	
Serum					
CRP (mg/L)	**173.2**	3.5	<0.3	**46.6**	0–5
Creatine-kinase (U/L)	**8.881**	**4.699**	**24.484**	**213.932**	0–200
Creatinine (μmol/L)	**221**	**528**	22	34	35–100
Urea (mmol/L)	**29**.**2**	**23**.**3**	3.6	**15**.**5s**	1.8–6.4
LDH (U/L)	**3.217**	**17.905**	**6.243**	216	<248
ASAT (U/L)	**1.180**	**20.264**	**2.293**	**2.942**	<51
ALAT (U/L)	**274**	**10.779**	**1.563**	**2.991**	<39
Ferritin (μg/L)	**18.732**	**74.416**	12	**499**	25–300
Lactate (mmol/L)	1.2				<2.2
Hemoglobin (mg/L)	**4**.**3**	**4.9**	6.7	8.1	6.5–10
Leukocytes (10^9^/L)	**20**.**5**	**19.1**	8.3		4.0–14.0
Platelet count (10^9^/L)	**1.186**	**19**	**703**	232	150–450
PT (s)	**12**.**0**	**77**	10.9	**12**.**3**	9.7–11.9
aPTT (s)	**37**	**105**	<20	<20	22–29
Fibrinogen (g/L)	1.3	**0**.**3**			0.8–3.3
D-dimer (mg/L)	**>35.20**	**>35.20**			0–0.5
Urine					
Erytrocytes (#/μl)	193	<4	4	**>1,000**	
Protein/creatinine ratio	**179**.**4**	**1.116**	**81**.**8**	**28**.**5**	

CMV, Cytomegalovirus; CRP, C-reactive protein; LDH, lactate dehydrogenase; ASAT, aspartate aminotransferase; ALAT, alanine aminotransferase; PT, prothrombin time; aPTT, activated partial tromboplastin time; sec, seconds.

Bold: strongly abnormal serum levels.

### Case B

An 8-year-old girl with cerebral palsy, multifocal epilepsy of unknown etiology and developmental delay, was admitted to the pediatric intensive care unit (PICU) for respiratory insufficiency secondary to a convulsive status. Physical examination showed tachycardia, anuria and spontaneous mucosal bleeding and epistaxis. Serum levels of CK and creatinine were increased (Table 1). Also, liver failure was apparent with severely elevated transaminases levels, cholestasis and prolonged plasma coagulation tests (not due to vitamin K deficiency) and extreme ferritin levels (Table 1 and Figure 1). Urine analysis showed myoglobinuria and nephrotic range proteinuria. A PCR of nasopharyngeal swab was positive for Influenza A viral RNA. The girl was started on mechanical ventilation and continuous hemodiafiltration for AKI and liver failure. The girl was treated with daily plasmapheresis until kidney biopsy ruled out thrombotic microangiopathy. Other causes of liver failure and hyperferritinemia as a consequence of primary and secondary causes of hemophagocytic lymphohistiocytosis (no fever, cytopenia, or splenomegaly and normal soluble CD25 values [i.e., <2.400 U/ml]) had been excluded. The diagnosis was influenza A virus infection induced rhabdomyolysis, AKI and hepatitis complicated with progressive liver failure and disseminated intravascular coagulation. The patient was considered eligible for liver transplantation. Due to the severe complications and progressive hepatic encephalopathy, the girl passed away. Genetic testing by post-mortem WES and Single Nucleotide Polymorphism (SNP) array found no explanation for the etiology and outcome of her affliction.

### Case C

A 2-year-old girl was admitted for a 7-day history of fever, vomiting, neck pain, myalgia, muscle weakness and dark urine. Treatment with oral amoxicillin did not result in clinical improvement. Laboratory studies revealed increased CK levels, elevated liver blood tests, normal kidney function, and low plasma ferritin levels (Table 1). Enterovirus was detected in the feces and in a nasopharyngeal swab. The same nasopharyngeal swab tested also positive for parainfluenza virus type 3. She was admitted with a diagnosis of rhabdomyolysis and treated with hyperhydration followed by rapid normalization of all laboratory findings and clinical symptoms without development of AKI.

### Case D

A 14-year-old girl presented with fever for 5 days, myositis, muscle weakness and a sore throat. Urine color was dark. Upon admission, serum CK levels and liver transaminases were strongly increased (Table 1). Her kidney function was normal (Table 1 and Figure 1). Investigations into the origin of the rhabdomyolysis revealed a primary cytomegalovirus (CMV)-infection. The diagnosis was made based on CMV DNA load in blood positive with a concentration of 5.25 × 10^3^ U/L and positive CMV IgM), and exclusion of other viral or metabolic causes. Because of respiratory symptoms due to muscle weakness, she was admitted at the PICU for respiratory support. Additional testing for respiratory pathogen with multiplex PCR on a nasopharyngeal swab was negative. Following hyperhydration, laboratory abnormalities receded and her clinical condition improved.

## Discussion

In this small case series, we describe four pediatric patients with rhabdomyolysis secondary to a viral infection. Rhabdomyolysis is defined by an elevation of CK levels up to 5 times of normal (2). In the majority of pediatric patients rhabdomyolysis is usually mild, comparable to two of our cases (Cases C and D). Recent literature suggests that secondary acute kidney failure attributed to rhabdomyolysis in children is rare and only occurs in less than 5% (2, 5). Two of our cases, caused by influenza A infection (Cases A and B), demonstrate this complicated clinical course with rhabdomyolysis, acute kidney failure and, in one, also liver failure. Strikingly, both patients have marked hyperferritinemia, whereas ferritin levels are normal or only slightly increased in the milder cases.

Studies on the combination of virus-induced rhabdomyolysis and AKI in children are limited. Viruses known to induce rhabdomyolysis are influenza virus, adenovirus, respiratory syncytial virus, parainfluenza virus type 1, Ebstein Barr virus, coronavirus, enterovirus, rhinovirus and CMV (6). More recently, the SAS-CoV-2 omicron variant has also been associated with rhabdomyolysis in children (5). A recent study reported AKI in up to 45% of pediatric patients with rhabdomyolysis induced by Influenza A (5). Occurrence of AKI in non-virus -induced rhabdomyolysis is also reported. In all cases older children are more likely to develop AKI (6).

The pathophysiologic mechanism underlying rhabdomyolysis-induced AKI is likely to be multifactorial. Contributing factors are renal exposure to myoglobulin toxicity, direct viral injury to the kidney, renal hypoperfusion and/or disseminated intravascular coagulation (7). Accumulation of myoglobin in the kidney causes toxicity leading to heme-iron mediated stress, tubular death and inflammation (1). Myoglobin is detoxified into heme myoglobin and ferritin in the kidney. Next to its formation in the detoxification, ferritin by itself is also associated to rhabdomyolysis-induced AKI (1). The iron cascade is thought to be a critical initiator of oxidative stress and serum ferritin levels a predictor of AKI development (8). The predictive value of ferritin in severe acute kidney injury has previously been described (4).

In accordance with this hypothesis, the hyperferritinemia observed in Cases A and B may have played an important role in the development of AKI. In both cases, the strongly elevated serum ferritin levels are unexpected. Ferritin is an (semi) acute-phase reactant produced and released by macrophages, hepatocytes and proximal tubular renal cells (but not by muscle cells), when activated by inflammatory cytokines such as interleukin (IL)-1β and tumor necrosis factor (TNF)-α (9). As such, it is part of the innate immune response and may sequester iron to prevent microbial outgrowth and dissemination (10). In addition, ferritin may be induced to inactivate free iron released by oxidation reactions. A previous study describes the association between viremia, ferritin levels >1,000 ng/ml and increased risk of PICU admission and mortality (11). Viral infections are also common triggers of hemophagocytic lymphohistiocytosis and macrophage activation syndrome, characterized by hyperinflammation and hyperferritinemia (11). In Cases A and B these diagnoses are ruled out to cause the hyperferritinemia. Whether there is a specific association between influenza A virus and increased risk of hyperferritinemia compared to other viruses remains unclear and has not been described so far. A limitation of this case series is that it only includes two patients with severe outcome in virus-induced rhabdomyolysis. A larger cohort is needed to empower our hypothesis that hyperferritinemia may be a predictor for severe outcome in rhabdomyolysis. Another contributing cause for the hyperferritinemia in Case A and B besides the AKI may be the acute liver injury and in Case B the severe influenza A induced hepatitis which progressed to liver failure (12).

In summary, we describe four cases of children suffering from virus-induced rhabdomyolysis. In two cases with influenza A infection hyperferritinemia was observed and both developed acute kidney injury. The two other cases did present with rhabdomyolysis (and in one case with extremely elevated CK levels), but recovered completely and rapidly with supportive hyperhydration only. The exact tissue origin of the hyperferritinemia during the influenza A infection is unclear. However, the combination of rhabdomyolysis-mediated renal disease and liver injury may contribute to the extreme hyperferritinemia. Based on these cases our hypothesis is that in rhabdomyolysis, serum ferritin levels may predict the development of acute kidney injury and may be associated with an increased risk of mortality. Therefore, clinicians should be aware of the variation in outcome, severe complications of virus-induced rhabdomyolysis and the potential of ferritin as a possible predicting biomarker for severe outcome.

## Data Availability

The original contributions presented in the study are included in the article/Supplementary Material, further inquiries can be directed to the corresponding author.
